# Effect of Sn Addition on Microstructure and Corrosion Behavior of As-Extruded Mg–5Zn–4Al Alloy

**DOI:** 10.3390/ma12132069

**Published:** 2019-06-27

**Authors:** Jian Ding, Xin Liu, Yujiang Wang, Wei Huang, Bo Wang, Shicheng Wei, Xingchuan Xia, Yi Liang, Xianhua Chen, Fusheng Pan, Binshi Xu

**Affiliations:** 1 School of Material Science and Engineering, Hebei University of Technology, Tianjin 300130, China; 2 National Key Laboratory for Remanufacturing, Academy of Army Armored Forces, Beijing 100072, China; 3 College of Materials Science and Engineering, Chongqing University, Chongqing 400045, China

**Keywords:** Mg–5Zn–4Al, Sn, microstructure, corrosion property, anticorrosion mechanism

## Abstract

The effect of Sn addition on the microstructure and corrosion behavior of extruded Mg–5Zn–4Al–xSn (0, 0.5, 1, 2, and 3 wt %) alloys was investigated by optical microscopy (OM), field emission scanning electron microscopy (FE-SEM), transmission electron microscopy (TEM), X-ray diffraction (XRD), X-ray photoelectron spectroscopy (XPS), electrochemical measurements, and immersion tests. Microstructural results showed that the average grain size decreased to some degree and the amount of precipitates increased with the increasing amount of Sn. The extruded Mg–5Zn–4Al–xSn alloy mainly consisted of α-Mg, Mg_32_(Al,Zn)_49_, and Mg_2_Sn phases as the content of Sn was above 1 wt %. Electrochemical measurements indicated that the extruded Mg–5Zn–4Al–1Sn (ZAT541) alloy presented the best corrosion performances, with corrosion potential (E_corr_) and corrosion current density (I_corr_) values of −1.3309 V and 6.707 × 10^−6^ A·cm^−2^, respectively. Furthermore, the corrosion mechanism of Sn is discussed in detail.

## 1. Introduction

Magnesium alloys have been widely used in the aerospace and automobile industries, electronics, and various other fields because of their light weight, high strength/weight ratios, good thermal conductivity, and excellent electromagnetic shielding characteristics [1,2,3]. However, commercial applications have been limited due to their poor corrosion resistance, especially in aggressive chloride ion environments [4,5]. 

To improve their corrosion resistance, alloying [6,7,8], surface modification [9,10,11,12], heat treatment [13], and the plastic deformation process [14] have been applied. Among these methods, alloying is an effective way to improve anticorrosion properties. Thus, various elements, including Al, Zn, Ca, Sr, RE, Sn, and Si, have been added to Mg alloys to change their anticorrosion properties. Among these, RE, Al, Zn, and Sn show great advantages in terms of anticorrosion properties. RE is an element that has the potential to improve the corrosion resistance of Mg alloys [15,16]. However, the exploitation and refinement of rare earths can cause serious damage to renewable energy, which limits the wide application of RE [17]. Al and Zn are most commonly used in Mg alloys due to their grain refinement and solid solution strengthening effect [18,19]. However, there is much more microgalvanic corrosion as the concentration of Al and Zn increases, leading to poor corrosion resistance. Further, Sn has been attracting more and more attention because of its grain refinement and reduction of the hydrogen evolution rate of Mg alloys [20,21]. Therefore, it is necessary to find one low-cost alloy system to improve the corrosion resistance performance of Mg alloys.

In recent years, much more attention has been paid to Mg–Zn–Al–Sn (ZAT) alloys due to their low cost, high strength, and excellent plasticity both at room and elevated temperatures. It has been proved that the tensile strength and elongation of ZAT alloys can reach 300 MPa and 20% [22,23], respectively, which is superior to traditional ZM5 or ZK60 and comparable to Mg–RE alloys [24]. Although several studies have been done on the mechanical properties of ZAT alloys [22,23,25,26,27,28,29,30], few researchers have focused their attention on their corrosion resistance [31,32,33]. Further, the corrosion mechanism and corrosion product types of ZAT alloys are not yet clearly understood. Therefore, it is necessary to study the effect of Sn on the corrosion resistance of Mg–Zn–Al alloys.

In the present work, the microstructure evolution and corrosion behavior of as-extruded Mg–5Zn–4Al–xSn (0, 0.5, 1, 2, and 3 wt %) alloys were investigated systematically. The purpose was to explore the optimum Sn addition amount for the highest corrosion resistance of the Mg–5Zn–4Al system and to clarify the corrosion mechanism of Sn-containing Mg–Zn–Al alloys. The findings of this study will aid in the development of high-corrosion-resistance and low-cost Mg alloys.

## 2. Experimental Procedures

### 2.1. Sample Preparation

Mg ingot (99.95%), Zn ingot (99.99%), Al ingot (99.95%), and Sn (99.99%) were used for the matrix to prepare the Mg–5Zn–4Al–xSn alloy. Prior to melting, the materials were placed in an electric resistance furnace at 720 °C under the protection atmosphere of CO_2_ and SF_6_. Subsequently, the molten alloy was stirred for 10 min at 740 °C to remove slag inclusion and impurities, and the temperature was held for 20 min to homogenize the composition. The detailed preparation process was described previously [23,26]. The dimensions of the cast billet were 80 mm in diameter and 120 mm in length. The ingot composition was determined by inductively coupled plasma (ICP), as shown in Table 1. The result was consistent with the nominal mass percentage in alloys. The extrusion temperature was 300 °C, the extrusion ratio was 25:1, and the extrusion speed was 2 mm/s. It was necessary to preheat the ingots and extrude the molds before extrusion. Finally, the diameter of the extruded bar gained was 16 mm. 

### 2.2. Microstructural Characterization

All of the metallographic samples were cut from the center of the ingots perpendicular to the extrusion direction (ED) to ensure comparability. Optical microscopy (OM) (SMZ800, Nikon, Tokyo, Japan), field emission scanning electron microscopy (FE-SEM) (Nava Nano FE-SEM450/650, Eindhoven, Holland, accelerating voltage of 20 kV) equipped with an X-Max 80 energy-dispersive X-ray spectrometer (EDS, Horiba, Tokyo, Japan), and transmission electron microscopy (TEM) (LI-BRA200, Oberkochen, Germany) were used for microstructure observation. Prior to imaging for OM and SEM observation, the test samples were ground to 1500 grit by SiC papers, mechanically polished using alcohol, and dried in cold air. Subsequently, the specimens were etched with a mixture of 5 g picric acid, 40 mL ethanol, 5 mL acetic acid, and 10 mL deionized water. Image-Pro-Plus software (IPP 6.0, Media Cybernetics, Washington, DC, USA) was used for average grain size measurement and at least five optical micrographs were counted. X-ray diffraction (XRD) (D/MAX-2500PC, Rigaku, Tokyo, Japan) was used to analyze the phase by using Cu kα. The diffraction angle was set from 20° to 80° with a scanning rate of 2°/min. 

### 2.3. Electrochemical and Immersion Tests

The corrosion behavior of the extruded Mg–5Zn–4Al–xSn alloys was measured by immersion tests, potentiodynamic polarization, and electrochemical impedance spectroscopy (EIS). Electrochemical behavior measurements of all specimens were carried out on an electrochemical workstation (IM6ex, Zahner, Kronach, Germany) under 3.5 wt % NaCl solution at room temperature. All samples were cylindrical with a diameter of 16 mm and a height of 10 mm. The effective area was about 2.01 cm^2^ (Φ16 mm). The classical three-electrode cell (the measured sample was the working electrode, a platinum plate was the auxiliary electrode, and the reference electrode was a Ag/AgCl saturated calomel electrode) was adopted for the electrochemical test. Before the test, specimens were immersed into 3.5 wt % NaCl solution for 300 s to obtain a stable open-circuit potential. Potentiodynamic polarization tests were carried out from −0.3 to +0.6 V versus open-circuit potential with a scanning rate of 1 mV/s. The polarization curves were fitted using CView-2 (Scribner, Southern Pines, NC, USA). EIS measurements of the as-prepared alloys were performed from 10^−2^ to 10^5^ Hz with 10 mV sinusoidal perturbation amplitude and the results were fitted by Zview 2.0 software (Scribner). All experiments were performed more than three times to ensure repeatability and consistency. 

### 2.4. Corrosion Morphology and Products

In order to describe the evolution of corrosion behavior and the origin of pitting corrosion more clearly, immersion tests were carried out. The polished specimens were immersed in electrolytes from 10 min to 1 h, then washed with deionized water and cleaned with alcohol immediately. To investigate the initiation and propagation of corrosion, the surface corrosion morphology was recorded by the abovementioned SEM equipped with EDS. Microscopic corrosion products were examined by X-ray photoelectron spectroscopy (XPS) (Thermo Fisher Scientific, Waltham, MA, USA) after immersion in 3.5 wt % NaCl solution for 48 h.

## 3. Results and Discussion 

### 3.1. Microstructure

**Figure 1:**
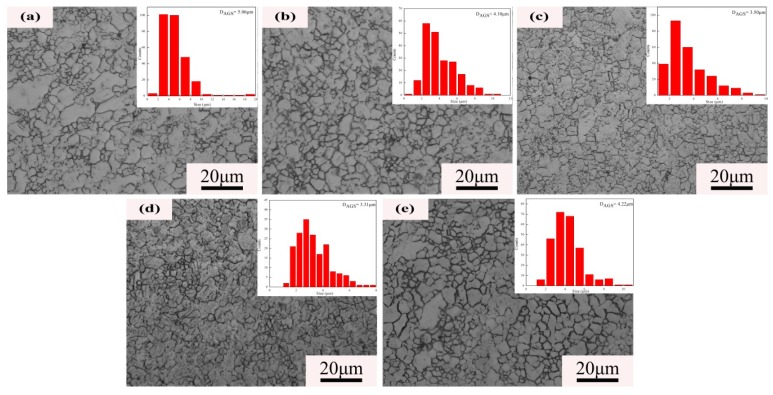
Optical microscopy (OM) of extruded Mg–5Zn–4Al–xSn alloys: (**a**) ZAT540, (**b**) ZAT540.5, (**c**) ZAT541, (**d**) ZAT542, and (**e**) ZAT543.

Figure 1 illustrates the microstructure of extruded Mg–5Zn–4Al–xSn (ZAT54x) alloys with different Sn addition amounts. The average grain size decreased as the Sn content increased to 2 wt %, and the average grain size increased as the content reached 3 wt %. Statistical results showed that the average grain sizes of ZAT540, ZAT540.5, ZAT541, ZAT542, and ZAT543 alloys were 5.06 ± 2.31, 4.1 ± 1.20, 3.5 ± 1.63, 3.31 ± 1.58, and 4.22 ± 2.05 μm, respectively. This means that Sn can be used as a grain refiner for Mg alloys [28]. Obviously, the grain size of the ZAT54x alloys was extremely inhomogeneous and was mainly distributed in the range of 0.9–20 μm due to the incompletely dynamic recrystallization during the extrusion process.

**Figure 2:**
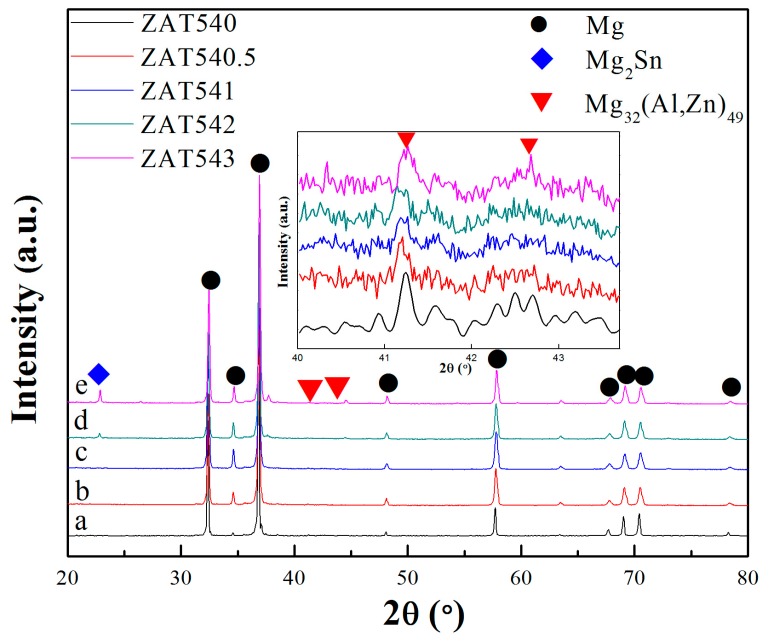
X-ray diffraction (XRD) patterns of extruded Mg–5Zn–4Al–xSn alloys: (**a**) ZAT540, (**b**) ZAT540.5, (**c**) ZAT541, (**d**) ZAT542, and (**e**) ZAT543.

XRD results (Figure 2) showed that only α-Mg and Mg_32_(Al,Zn)_49_ phases appeared where the Sn content was less than 2 wt %, while for the alloys with Sn contents of 2 wt % (ZAT542) and 3 wt % (ZAT543), Mg_2_Sn diffraction peaks appeared and the diffraction peak intensities were enhanced as the Sn content increased. Formation of the Mg_2_Sn phase was primarily due to the higher electronegativity differences between Mg–Sn (0.65) than that of Sn–Al (0.35) and Sn–Zn (0.31) [34].

**Figure 3:**
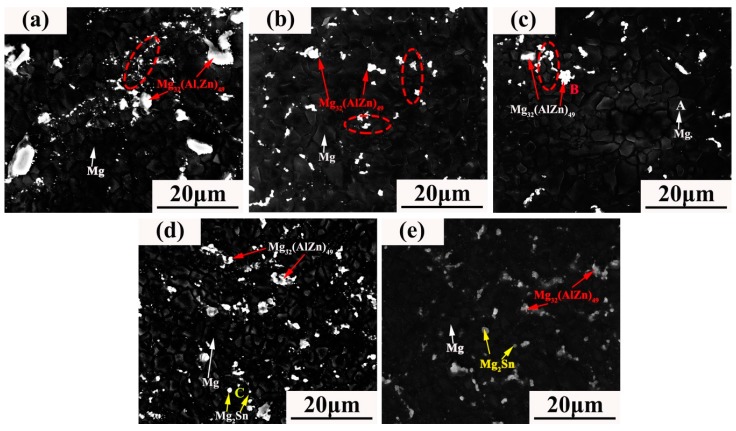
SEM images of extruded Mg–5Zn–4Al–xSn alloys: (**a**) ZAT540, (**b**) ZAT540.5, (**c**) ZAT541, (**d**) ZAT542, and (**e**) ZAT543.

Figure 3 shows the microstructure evolution of ZAT54x alloys. A large number of precipitates were distributed in the grain boundaries and a small number of second phases in grain interiors. Meanwhile, the number of precipitated phases increased gradually as the amount of Sn increased. One interesting thing is that the grains near the precipitations were smaller compared with those of around the nonprecipitations (marked with red dotted lines), which implies that uniform recrystallization was formed. The reason was that the deformation zone near the hard particles became the preferred nucleation point for recrystallization due to the high dislocation density and large directional gradient during extrusion [35].

In terms of SEM images, there were two different kinds of morphologies: bright flake-like of larger size (marked as B) and bright globular-shaped particles (marked as C). According to the EDS results shown in Table 2, B is rich in Mg, Zn, and Al. Combined with XRD, B was deduced as being the Mg_32_(Al,Zn)_49_ phase. C particles were determined by EDS to contain about 73.90 at. % Mg and 26.10 at. % Sn, and they may be the Mg_2_Sn phase. 

**Figure 4:**
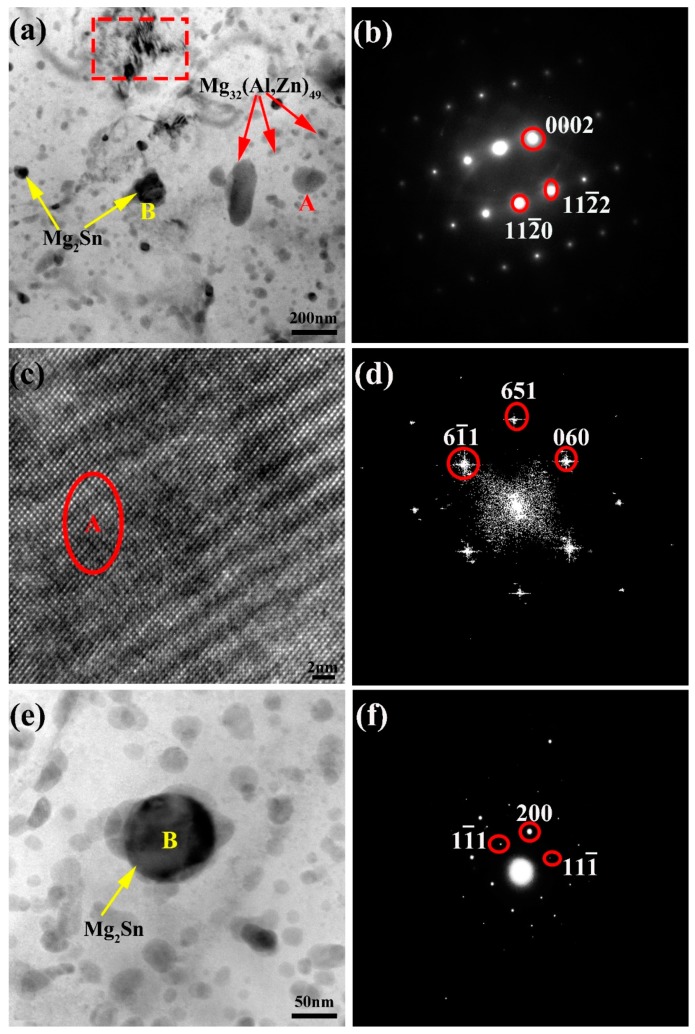
(**a**) Transmission electron microscopy (TEM) images of extruded ZAT541 alloy. (**b**) The selected area electron diffraction (SAED) of matrix. (**c**) High-resolution episcopic microscopy (HREM) image of precipitation A. (**d**) Fast Fourier transform (FFT) pattern obtained from A. (**e**) The high-magnification TEM morphology of a B particle. (**f**) The SAED of B.

To clarify the composition of the precipitations in the extruded ZAT54x alloys, TEM detections on the ZAT541 alloy were carried out (Figure 4). There were much more secondary precipitates, large numbers of light-gray precipitations (marked as A), and a few black spherical particles (of finer size of about 10–50 nm in the grain boundary, marked as B) homogeneously in matrix. Moreover, it can be seen in Figure 4a that high-density dislocations were distributed at the grain boundary (red rectangle); further analysis is shown in Figure 4b–f. Figure 4b shows the selected area electron diffraction (SAED) of the matrix with a hexagonal close-packed structure (HCP). Figure 4c,d display the high-resolution episcopic microscopy (HREM) image and fast Fourier transform (FFT) pattern of A. According to our analysis, A can be identified as the Mg_32_(Al,Zn)_49_ phase with the space group Im3(204). This was consistent with the results from SEM and XRD. A typical spherical B particle is shown in Figure 4e. The SAED pattern of B was confirmed by <011> zone axes of Mg_2_Sn (a = 0.68 nm) with a face-centered cubic (FCC) structure, as shown in Figure 4f. The Mg_2_Sn phase could not be detected by SEM and XRD of the ZAT541 alloy because of its smaller dimension and quantity. Further, the Mg_2_Sn phase has the orientation relationship with the matrix wherein [2 −1 −1 0] Mg // [0 1 1] Mg_2_Sn, (0 0 0 2) Mg // (1 −1 1) Mg_2_Sn [28].

From the microstructure analysis, the grain size decreased as the content of Sn increased (Figure 1; Figure 3). The reasons were that hot extrusion can cause lattice deformation and increase the density of dislocations and vacancies, causing an increase of storage strain energy [36]. High-density dislocations during extrusion are shown in Figure 4a. Strain energy can be used as a driving force for grain nucleation and growth during recrystallization. Further, finer Mg_2_Sn phases distributed at grain boundaries (Figure 4a) can act as nucleation particles to promote dynamic recrystallization by the particle-stimulated nucleation (PSN) mechanism [37]. Furthermore, the fine precipitates at the grain boundary can also inhibit grain growth by the boundary pinning effect [29]. When the Sn content was 3 wt %, coarse Mg_2_Sn and Mg_32_(Al,Zn)_49_ precipitates formed in matrix, resulting in larger grain sizes due to grain the boundary pinning effect being reduced. Remarkably, the distribution of grain sizes during extrusion was extremely uneven (Figure 1). This was because the large number of precipitations increased the dislocation density and changed the uniformity of slip, leading to incomplete dynamic recrystallization during extrusion [30].

### 3.2. Potentiodynamic Polarization Test

**Figure 5:**
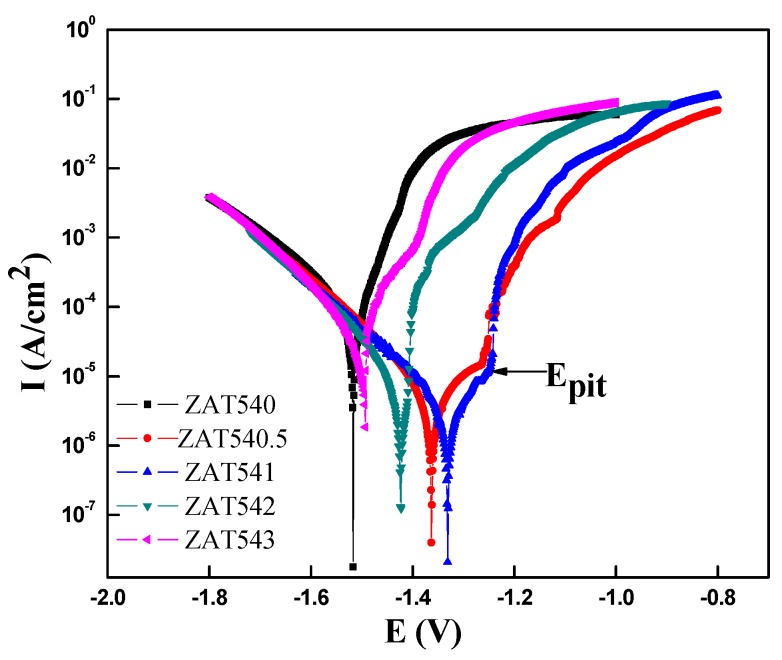
Polarization curves of extruded ZAT54x alloys in 3.5 wt. % NaCl solution at room temperature.

The typical potentiondynamic polarization curves of the as-extruded alloys were tested in 3.5 wt % NaCl solution at room temperature, as displayed in Figure 5. There was the same trend in polarization curves with different Sn contents. The cathode was controlled by the reaction of hydrogen evolution and the anode dissolved by matrix as the applied anodic potential increased. This showed that the corrosion potential first increased and then decreased as the Sn content increased. When the Sn content was 1 wt %, the corrosion potential of the polarization curve was the most positive. As it is clear, the corrosion potential of the extruded ZAT541 alloy shifted about 200 mV to the positive direction compared with the ZAT543 and ZAT540 alloys. In addition, the anode current branches of the polarization curve moved to a relatively low value in the ZAT541 alloy, which indicates the reduction of the anodic dissolution rate and the corrosion protection of the alloy.

There was obvious passivation behavior in the extruded ZAT54x alloys due to the formation of an oxide film on the alloys’ surface. However, the anticorrosion properties of oxide film are undesirable because the anodic current densities rise slowly with the increasing anodic potential below the breakdown potential (E_pit_ as marked on the curves) due to the porous structure of oxide films [38].

The potentiodynamic polarization curves were analyzed by the cathodic Tafel extrapolation technique due to the negative difference effect (NDE) in the anodic branch for Mg alloys [39,40]. The fitting parameters of polarization, including corrosion potential (E_corr_), corrosion current density (I_corr_), and cathodic Tafel slope B_c_, are given in the Table 3. The E_corr_ values of the ZAT540, ZAT540.5, ZAT541, ZAT542, and ZAT543 alloys were −1.5173, −1.3628, −1.3309, −1.4235, and −1.4941 V, respectively. Their I_corr_ values were 6.6571 × 10^−5^, 9.4157 × 10^−6^, 6.7071 × 10^−6^, 1.1387 × 10^−5^, and 2.5085 × 10^−5^ A·cm^−2^, respectively. From the above results, the ZAT541 alloy showed excellent corrosion resistance. The reason was that solutionized Sn can inhibit the hydrogen evolution reaction at the cathodic branch of polarization curves due to the high hydrogen overpotential as the content of Sn was less than 2 wt %, resulting in an increase in the corrosion resistance of the alloy [41]. Further, many more Mg_2_Sn precipitates were distributed in the grain boundary, which increased the probability of pitting and the corrosion propagation rate, accelerating the dissolution of the matrix. 

### 3.3. Electrochemical Impedance Spectroscopy 

**Figure 6:**
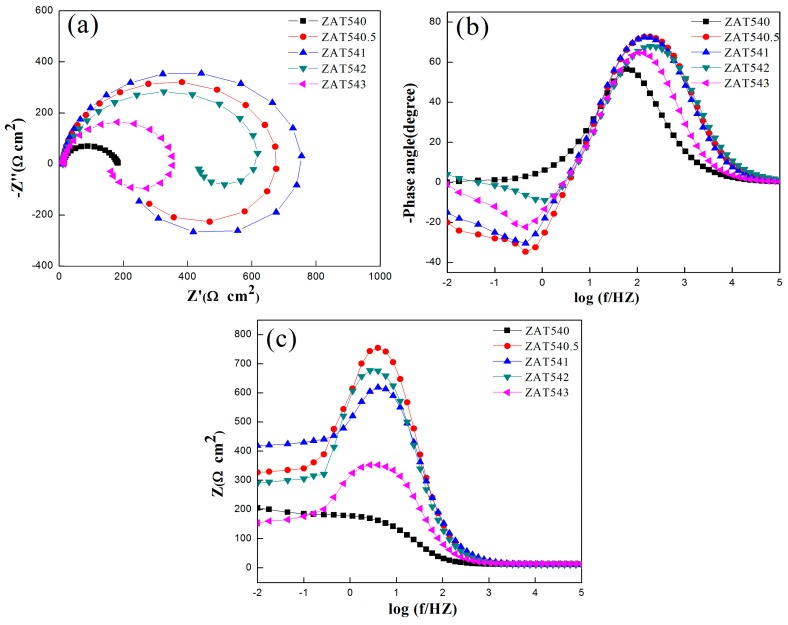
(**a**) Nyquist plots of ZAT54x alloys. (**b**) Bode plots of phase angle vs frequency for ZAT54x alloys. (**c**) Bode plots of impedance vs frequency for ZAT54x alloys.

EIS results of all the samples are shown in Figure 6. As shown in Figure 6a, the Nyquist plots of the alloys consisted of a bigger semicircular loop at high frequencies, which we attributed to capacitance characteristics, and a semicircular loop at lower frequencies caused by the inductance of the alloys. Normally, the diameter of the capacitor plays an important role in evaluating the anticorrosion properties of alloys [42]. In the case of these specimens, the larger diameter, the better corrosion resistance of the alloy. Therefore, the extruded ZAT541 alloy showed the best anticorrosion properties. Figure 6b displays the Bode phase plots versus frequency exposed in 3.5 wt % NaCl solution. All alloys displayed two time constants, including a crest at high frequency and a wave trough at low frequency. It was a one-to-one match between the high-frequency capacitor loop and low-frequency inductance loop in the Nyquist plots. The deviation of the phase angle from 90° showed a facile relative charge transfer during corrosion [43]. It can be seen that the extruded ZAT541 alloy with a higher phase angle (−72.4°) at high frequency resulted in better corrosion resistance. Figure 6c shows the Bode impedance plots of the specimens. The anticorrosion ability of alloys is usually evaluated by the magnitude of impedance modulus at low frequencies (|Z|_0.01HZ_). The impedance modulus of ZAT540, ZAT540.5, ZAT541, ZAT542, and ZAT543 alloys was 205, 327, 419, 290, and 154 Ω cm^2^, respectively. This implies that the ZAT541 alloy exhibited the strongest anticorrosion capability. All EIS results coincided with the polarization curve analyses.

**Figure 7:**
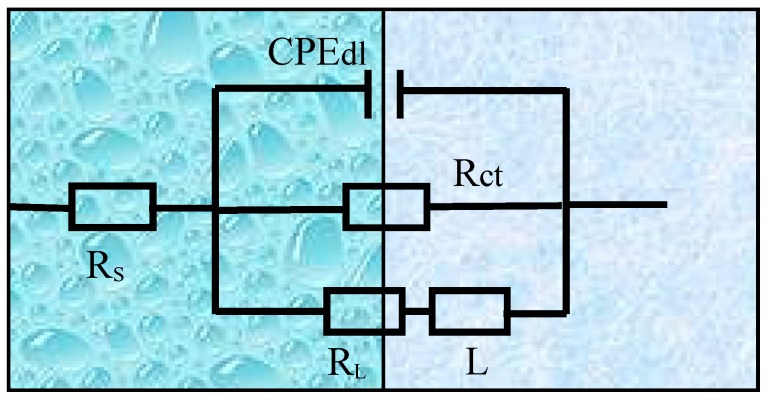
Equivalent circuit of electrochemical impedance spectroscopy (EIS) for extruded ZAT54x alloys.

From the above results, the equivalent electrical circuits were obtained by Zview 2.0 software. Figure 7 illustrates the equivalent circuit model, and fitting parameter data are shown in Table 4. R_s_ indicates the electrolyte solution resistance between the working and reference electrodes. CPE_dl_ represents a constant phase element to compensate for the nonhomogeneity of the capacitor system, which is related to the electric double layer between the dielectric solution and the α-Mg matrix at the interface. R_ct_ is the charge transfer resistance. L and R_L_ stand for inductance and inductance resistance, respectively. The appearance of L means pitting corrosion of the alloy surface.

From the fitting data, R_ct_ was 317.5, 647.2, 681.6, 635.4, and 345.6 Ω cm^2^, respectively. The R_ct_ was the largest with the addition of 1 wt % Sn, reflecting the minimum dissolution rate of the substrate. However, excessive Sn addition caused the corrosion resistance to decrease. Based on impedance spectra and equivalent circuit fitting, the corrosion kinetics of the extruded ZAT54x alloys is described below. The interface between alloy surfaces and NaCl solution was formed the CPE, and there was a charge transfer between two electrodes as the alloys were immersed in the NaCl solution. Microgalvanic corrosion occurred firstly on the matrix adjoined to the second phases due to the difference of potential. Because of the precipitates (Mg_32_(Al,Zn)_49_ and Mg_2_Sn) with high surface energy and activity, it could accelerate the corrosion rate and corrosion reactivity of the matrix. With the dissolution of the matrix, passivation formed on the surface of the alloys resulted in the formation of a corrosion product layer (Figure 5). However, it was loose and porous and could not protect the matrix effectively. Therefore, Cl^−^ permeated the matrix through the protective film, leading to pitting corrosion.

### 3.4. Corrosion Behavior 

**Figure 8:**
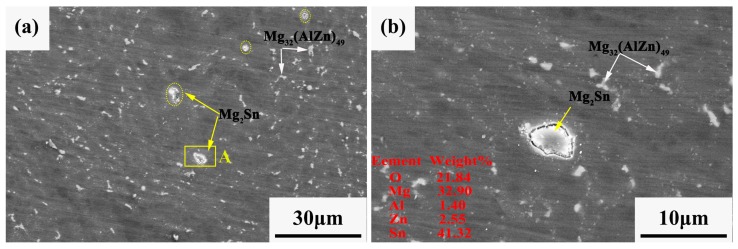
(**a**) Initiation corrosion sites of ZAT543 alloy immersed in 3.5 wt % NaCl for 10 min; (**b**) high-magnification morphology of region A.

The microstructure of the extruded ZAT543 alloy was immersed in 3.5 wt % NaCl solution for 10 min, as shown in Figure 8. The evolution and origination of pitting corrosion in the ZAT543 alloy was easier due to the large number of precipitations. Figure 8a shows the alloy pitting characteristics at low magnification. It can be observed that white corrosion products were adjacent to Mg_2_Sn metallic compounds (marked as region A), which appeared to fall off and cause corrosion phenomenon around the matrix severely, while the matrix around the Mg_32_(Al,Zn)_49_ phase was uncorroded. Figure 8b is the high-magnification pitting morphology of region A, and corresponding EDS are inserted in the image. It shows that pitting corrosion preferentially occurred at Mg_2_Sn precipitation due to the noble potential, and Mg_32_(Al,Zn)_49_ can be a corrosion-resistant phase. 

**Figure 9:**
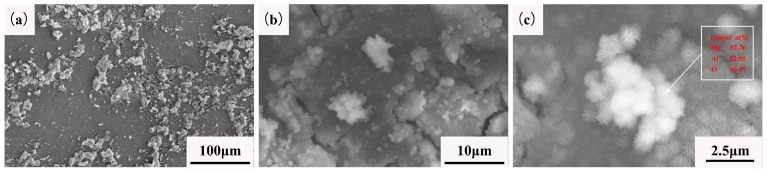
(**a**) Corrosion morphology of ZAT543 alloy immersed in 3.5 wt% NaCl for 1 h; (**b**) high-magnification corrosion morphology; (**c**) corrosion products of ZAT543 alloy.

The morphology of the extruded ZAT543 alloy was immersed in 3.5 wt % NaCl solution for 1 h, as shown in Figure 9. Figure 9a shows that typical filiform corrosion and cracks appeared in the contact between the matrix and the corrosion products on the surface of ZAT543. A lamellar gray corrosion product layer adhered to the surface of the alloys with the white needle-like globular product on the top layers (Figure 9b). The reason for the cracks in the corrosion product layer is as follows. Firstly, pitting corrosion occurred at different locations due to various precipitates existing in the alloys. Corrosion products that formed at different locations collided to crack when they touched each other due to the different growth rates. Then, MgO of the cubic crystal systems transformed into Mg(OH)_2_ of the hexagonal crystal system, leading to the volume of products expanding twice as much as before. Lastly, a large amount of the Cl^−^ loose corrosion product layer resulted in peeling as Cl^−^ permeated into the matrix through porous film layers. As shown in Figure 9c, corrosion products of the alloy surface determined by EDS contained about 32.76 at. % Mg, 2.95 at. % Al, and 64.29 at. % O. There was no H because EDS could not detect it. This means that the needle-like white corrosion products were Mg(OH)_2_.

### 3.5. Corrosion Products Analysis 

**Figure 10:**
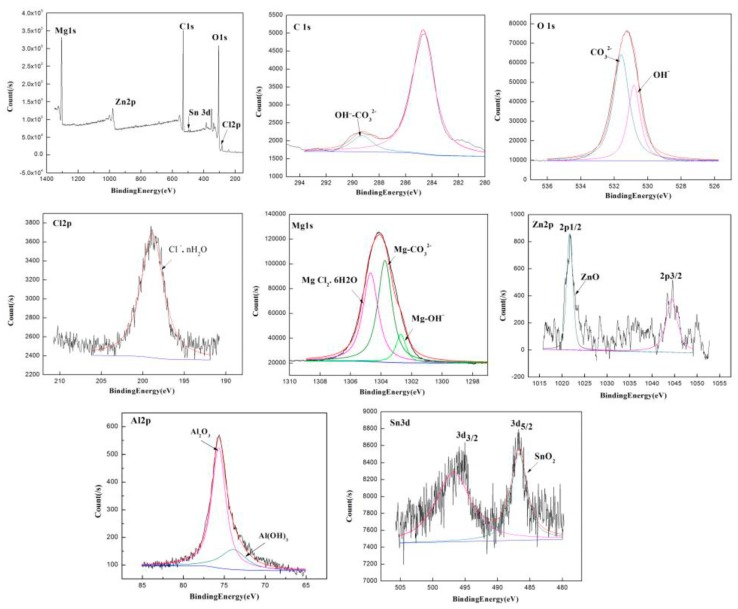
X-ray photoelectron spectroscopy (XPS) analysis results of ZAT543 alloy immersed in 3.5 wt % NaCl for 48 h.

The corrosion products of the extruded ZAT543 alloy immersed in 3.5 wt % NaCl for 48 h were detected by XPS, and the result is shown in Figure 10. There was C, O, Cl, Mg, Zn, Al, and Sn in the corrosion products of the ZAT543 alloy. As for C 1s, the binding energy was approximately 284.5 and 289.5 eV, corresponding to adventitious C and CO_3_^2−^. As for O 1s, there were two peaks corresponding to OH^−^ and O^2−^, which proved the existence of hydroxide and oxide. As for Cl 2p, the peak at 198.5 eV originated from Cl^−^·nH_2_O, which means that there were basic chloridion compounds in the corrosion products. In the case of the Mg 1s spectrum, the peaks at approximately 102.7, 1303.8, and 1304.8 eV originated from Mg(OH)_2_, MgCO_3_, and MgCl_2_·6H_2_O, respectively. For Zn 2p, there were two peaks corresponding to Zn 2p1/2 and Zn 2p3/2. This means that there was ZnO in the corrosion products. For the Al 2p spectrum, the binding energy was approximately 75.6 and 73.9 eV, corresponding to Al_2_O_3_ and Al(OH)_3_. For Sn 3d, there were two peaks in the Sn 3d spectrum consistent with Sn 3d3/2 at 494.9 eV and Sn 3d5/2 at 486.4 eV. This implies there was SnO_2_ in the corrosion products. Thus, the surface of the corrosion layer mainly consisted of a large amount of Mg(OH)_2_, MgCO_3_, and MgCl_2_·6H_2_O and a little Al_2_O_3_, Al(OH)_3_, ZnO, and SnO_2_.

From the corrosion behavior of the extruded alloys, the corrosion process can be divided into initiation, propagation, passivation, corrosion, and repassivation. At the initial stage of corrosion, severe microgalvanic corrosion occurred between the Mg_2_Sn and Mg matrix (Figure 8) on account of the stronger cathode effect and the potential difference with the matrix [41]. Pitting originated in the Mg_2_Sn phase. Meanwhile, the Mg matrix transferred to Mg^2+^ and H_2_O transferred to OH^−^, which was accompanied H_2_ release. Some Mg^2+^ combined with OH^−^ to form Mg(OH)_2_. The reaction process can be described by Equation (1):

(1) $$Mg+2H_{2}O=Mg{\left(OH\right)}_{2}+H_{2}↑.$$

Therefore, the galvanic effect around Mg_2_Sn precipitation dominated in the initial corrosion stage, which accelerated alloy corrosion. From the x E-PH diagram of the Sn–H_2_O diagram [44], tin hydride formation reaction may have occurred by Equation (2):

(2) $$2Mg+Sn+H_{2}O=SnH_{4}+2Mg{\left(OH\right)}_{2}.$$

At this time, Sn adsorbed H atoms, reducing the rate of hydrogen evolution of alloys. With the reaction going on, Sn enriched on the surface of the alloy and the reaction occurred by Equation (3) [45]:

(3) $$SnH_{4}+2H_{2}O=SnO_{2}+3H_{2}↑.$$

The corrosion products of Mg(OH)_2_ and SnO_2_ film (Figure 10) adhered to second phases, inhibiting the cathode effect of Mg_2_Sn. With the continuous invasion of Cl^−^, Mg^2+^ combined with Cl^−^ and H_2_O to form MgCl_2_·6H_2_O corrosion products, which are soluble in aqueous solution. Meanwhile, more galvanic corrosion formed and the hydrogen evolution rate improved greatly due to the large number of Mg_32_(Al,Zn)_49_ phases in the alloys (Figure 3). Pitting corrosion transformed into filiform corrosion (Figure 9a) and further transformed to overall corrosion. The corrosion rate was inhibited due to the coverage of corrosion products such as Mg(OH)_2_, MgCO_3_, Al_2_O_3_, Al(OH)_3_, ZnO, and SnO_2_ (Figure 9b and Figure 10) in the middle stage of corrosion. However, the protective film was porous, loose, and not compact, and Cl^−^ could penetrate the film into the fresh alloys’ surface and promote further acceleration of corrosion.

If the surface of the alloys can be repassivated, the corrosion resistance can be improved effectively. Soluble Sn and Al in the matrix deposit on the surface of the alloys, and a dense and stable oxide film (Al_2_O_3_, SnO_2_) is formed. The stable passivation film protects the substrate from further corrosion [32,46]. However, the overall corrosion rate of the alloys depends on the quantity of precipitations. A large number of Mg_2_Sn in the extruded ZAT543 alloy increased pitting sites significantly and promoted the release rate of H_2_, accelerating matrix dissolution. The accelerated H_2_ evolution can undermine the passivation film, losing its protective effect [28]. 

The extruded ZAT541 alloy showed the best corrosion resistance. Due to nonobvious grain size changes, the influence on corrosion was not considered. The excellent corrosion resistance could be attributed to the following reasons. On the one hand, solutionized Sn in the Mg matrix reduced the rate of hydrogen evolution due to the high hydrogen overpotential, resulting in a low dissolution rate of the matrix. On the other hand, a large quantity of precipitations distributed uniformly on the matrix, resulting in the randomness and homogeneity of the galvanic corrosion.

## 4. Conclusions

The microstructure and corrosion behavior of extruded Mg–5Zn–4Al–xSn alloys were studied. The main conclusions are as follows.

(1) The microstructure was different as more Sn was added. The as-extruded ZAT54x alloys consisted of α-Mg, Mg_32_(Al,Zn)_49_, and Mg_2_Sn phases. As the addition of Sn increased, grain refinement was achieved and the volume fraction of the Mg_2_Sn phase increased.

(2) The distribution of the second phases was uneven. They could act as cathodes to accelerate the corrosion of the matrix. Mg_2_Sn increased the possibility of pitting corrosion, the propagation of the corrosion rate, and the dissolution of the matrix.

(3) Through potentiondynamic polarization curves, EIS, and immersion tests, the addition of Sn was better for improving the anticorrosion properties of Mg–Zn–Al alloys by solution strengthening. The extruded ZAT541 alloy exhibited optimal corrosion resistance, for which the E_corr_ and I_corr_ values were −1.3309 V and 6.707 × 10^−6^ A·cm^−2^, respectively.

## Figures and Tables

**Table 1:** Composition analysis of cast Mg–5Zn–4Al–xSn (ZAT54x) alloys.

Alloy	Nominal Composition	Analyzed Composition (wt %)			
		Zn	Al	Sn	Mg
ZAT540	Mg–5Zn–4Al–0Sn	5.02	3.52	0.00	Bal
ZAT540.5	Mg–5Zn–4Al–0.5Sn	5.00	3.50	0.51	Bal
ZAT541	Mg–5Zn–4Al–1Sn	5.01	3.56	1.03	Bal
ZAT542	Mg–5Zn–4Al–2Sn	5.00	3.51	2.00	Bal
ZAT543	Mg–5Zn–4Al–3Sn	5.02	3.54	3.01	Bal

**Table 2:** Energy-dispersive X-ray spectroscopy (EDS) detection of extruded ZAT541 and ZAT542 alloys.

Position	Composition (at. %)			
	Mg	Zn	Al	Sn
A	90.88	05.30	03.82	0
B	62.55	19.17	18.28	0
C	73.90	0	0	26.10

**Table 3:** Potentiodynamic polarization test results of extruded Mg–5Zn–4Al–xSn alloys in 3.5 wt % NaCl solution at room temperature.

Sample	E_corr_ (V)	I_corr_ (A·cm^−2^)	B_c_ (mV/dec)
ZAT540	−1.5173	6.6571 × 10^−5^	130
ZAT540.5	−1.3628	9.4157 × 10^−6^	172
ZAT541	−1.3309	6.7071 × 10^−6^	203
ZAT542	−1.4235	1.1387 × 10^−5^	139
ZAT543	−1.4941	2.5085 × 10^−^^5^	126

**Table 4:** EIS fitting results of extruded Mg–5Zn–4Al–xSn alloys.

Alloys	Rs (Ω cm^2^)	CPE_dl_		R_ct_ (Ω cm^2^)	L (H cm^−2^)	R_L_ (Ω cm^2^)
		Q (S^n^ Ω^−1^ cm^−2^)	n			
ZAT540	11.53	1.481 × 10^−5^	0.9417	317.5	153.7	183.0
ZAT540.5	11.38	1.325 × 10^−5^	0.9623	647.2	167.5	261.4
ZAT541	11.22	1.505 × 10^−5^	0.9653	681.6	217.9	299.7
ZAT542	11.71	1.64 × 10^−5^	0.9195	635.4	172.4	215.4
ZAT543	14.39	2.38 × 10^−5^	0.9697	345.6	143.5	243.8
